# Association of physical therapy techniques can improve pain and urinary symptoms outcomes in women with bladder pain syndrome. A randomized controlled trial

**DOI:** 10.1590/S1677-5538.IBJU.2022.0056

**Published:** 2022-05-18

**Authors:** Claudia Rosenblatt Hacad, Marcos Lucon, Suehellen Anne Rocha Milhomem, Homero Bruschini, Clarice Tanaka

**Affiliations:** 1 Faculdade de Medicina da Universidade de São Paulo Divisão de Fisioterapia São Paulo SP Brasil Divisão de Fisioterapia, Faculdade de Medicina da Universidade de São Paulo - FMUSP, São Paulo, SP, Brasil; 2 Faculdade de Medicina da Universidade de São Paulo Divisão de Urologia São Paulo SP Brasil Divisão de Urologia, Faculdade de Medicina da Universidade de São Paulo - FMUSP, São Paulo, SP, Brasil

**Keywords:** Cystitis, Interstitial, Physical Therapy Modalities, Postural Balance

## Abstract

**Purpose:**

to verify the effects of biofeedback (BF) and manual therapy (MT) associated with transcutaneous electrical nerve stimulation (TENS) or postural exercises (PE) in the treatment of bladder pain syndrome (BPS) in women regarding pain and urinary symptoms.

**Materials and Methods:**

a parallel-randomized controlled trial was conducted in BPS patients diagnosed according to NIH clinical criteria. Two specialized physiotherapists applied demographic and validated questionnaires of perineal and suprapubic pain (VAS), urinary symptoms and problems (ICSI and ICPI) and sexual function (FSFI) and a physical assessment was made to identify myofascial trigger points. Thirty-one women, mean age 51.8 ± 10.9 were randomized in three groups of treatment consisting of ten weekly sessions of BF and MT (Conventional group); BF, MT, and TENS (TENS group); and BF, MT, and PE (Postural group).

**Results:**

Postural group improved perineal and suprapubic pain after treatment (p<0.001 and p=0.001, respectively), and the suprapubic pain improvement remained persistent at 3 months of follow up (p=0.001). Postural group improved urinary symptoms and problems after treatment (p<0.001 and p=0.005, respectively) and during follow up (p<0.001 and p=0.001).

**Conclusions:**

Biofeedback and manual therapy associated with postural exercises showed a significant improvement in perineal and suprapubic pain and urinary symptoms after treatment and during follow-up. Both results suggest a possible role for the use of this physiotherapy technique to treat BPS patients. Longer follow-up and a larger number of patients are necessary to confirm these conclusions.

## INTRODUCTION

Bladder pain syndrome (BPS) is defined as the presence of pain, pressure and discomfort in the pelvis, perineum, and genitalia for more than 6 months with at least one lower urinary tract symptom, such as frequency, urgency, or nocturia, in the absence of urinary infection or other pathology. This condition can be associated with pelvic floor disorders and gynecological and gastrointestinal symptoms and has a significant impact on sexual activity and overall quality of life (1, 2).

Considerable variability in treatment recommendations is noted in the main BPS guidelines. Based on phenotype classification, the treatment should be multidisciplinary and include behavioral, physical and psychological approaches (1, 3). Referring to the tenderness phenotype, women with BPS can present pelvic floor myofascial pain characterized by sensitivity during palpation and local or referred pain during the physical assessment (4, 5). An increase in muscle tension due to difficulties in relaxation that can generate myofascial trigger points has also been observed (5, 6). Trigger points can lead to pelvic floor dysfunction and consequently perineal pain and dyspareunia associated with urgency, frequency and nocturia (7, 8). Pelvic floor dysfunction can generate an increase in pelvic asymmetry and changes in diaphragmatic excursion, compromising respiratory and postural stability. Some evidence indicates that pelvic floor muscles contribute to respiratory and postural function (9).

The main guidelines (1-3) consider manual therapy and biofeedback valuable tools for the conservative treatment of BPS.

Manual therapy provides muscle fiber stretching, a decrease in muscle tension and trigger point release, restoring the normal movement amplitude and increasing body consciousness (10). Several studies show the efficacy of manual therapy to improve pain in women with BPS (11). The use of biofeedback with surface electrodes provides an indication of myoelectrical activity to modify the neuromuscular response and to acquire motor control improvement (12).

Despite the lack of recommendations in current guidelines, other noninvasive techniques, such as transcutaneous electro nerve stimulation (TENS) and postural exercises, may be associated with pain and urinary symptom treatment in women with BPS (13, 14).

TENS is used for acute and chronic pain through the placement of transcutaneous electrodes on the pain sites (15). The application is based on the gate control theory of pain, which suggests that a counter stimulation of the nervous system can modify the perception of pain. In the literature, women treated with high frequency, i.e., between 75-100 Hz, presented better pain effects and tolerability (13).

Postural exercises are used to improve musculoskeletal system function that can be compromised in the presence of visceral dysfunction in women with BPS. Musculoskeletal dysfunction is commonly demonstrated in women with self-reported chronic pelvic pain with the presence of iliac crest asymmetry and an increase in sensitivity in abdominal and pelvic floor muscles (14, 16-18). Long-lasting pain in the pelvic area generates neuroplasticity and contributes to disturbances in motor control and consequent changes in the activation performance of synergistic muscles, causing balance and gait disturbance, lack of coordination, a decrease in diaphragmatic excursion and an increase in tension in the pelvic area (19).

In this study, our hypothesis was that women with BPS presented musculoskeletal dysfunction, and we tested a different physiotherapy approach that has not being used. The reason for that understanding was the presence of refractory urinary and pain symptoms notwithstanding the physiotherapy conventional treatment, such as manual therapy and biofeedback. To test our hypothesis, we decided to add either TENS or postural exercises to the conventional treatment.

The objective of this study was to verify the effects of biofeedback (BF) and manual therapy (MT) associated with transcutaneous electrical nerve stimulation (TENS) or postural exercises (PE) in the treatment of bladder pain syndrome (BPS) in women regarding pain and urinary symptoms.

## MATERIALS AND METHODS

A parallel randomized controlled trial was conducted after approval from our Ethics Review Board (61595016.0.0000.0068) and registered at ClinicalTrials.gov (NCT03755375). Women with a diagnosis of BPS according to NIH clinical criteria (20) ≥ 18 years old were evaluated by the medical team of the Urology Clinic at the “Hospital das Clinicas of the University of Sao Paulo School of Medicine” and referred to a physical therapy outpatient clinic from February 2018 to December 2019. Women who presented symptoms of perineal and suprapubic pain, using painkillers, anticholinergics, antidepressants, and anticonvulsants for at least 6 months, and exhibited absence of urinary infection for at least 3 months for the initial assessment were included.

Women with positive urine culture, under actual or previous oncologic treatment, with systemic or neurological diseases that could compromise pelvic structures, with cognitive deficiency that compromised the understanding of the provided instructions and those who refused to participate in the study were excluded.

In the initial assessment, two specialized and trained physiotherapists applied a demographic questionnaire to identify the characteristics of the sample and validated questionnaires of perineal and suprapubic pain (Visual Analog Scale of Pain [VAS]) (21) to quantify the pain; urinary symptoms and problems (O’Leary-Sant - The Interstitial Cystitis Symptom and Problem Index) (22) to evaluate the presence of urgency, frequency, nocturia and to quantify how much these symptoms represent a problem to the patients, and the Female Sexual Function Index (FSFI) (23) to evaluate the impact on sexual life. Then, a physical assessment by the inspection and palpation of pelvic and perineal areas was made to identify myofascial trigger points.

After the assessment, participants were blinded randomized by a mask researcher using random.org and allocated into three groups of treatment (TENS, Postural and Conventional) held over 10 sessions once a week. All participants needed to attend the whole treatment to be included with a maximum delay of 2 weeks to start treatment.

Conventional group was treated with biofeedback for pelvic floor relaxation and manual therapy to release the tension in the suprapubic, pelvic, and intravaginal areas. The manual therapy consisted of a myofascial trigger point release maneuver using digital pressure and muscle fiber stretching in pain areas. Biofeedback consisted of pelvic floor muscle coordination and relaxation exercises using intravaginal probes. The training program was initiated with 10 fast contractions with 5 seconds of relaxation between them followed by 10 sustained contractions of 5 seconds with 10 seconds of relaxation between them. Finally, one minute of pelvic floor relaxation was performed.

TENS group was treated with biofeedback, manual therapy, and transcutaneous electrostimulation (TENS), a peripheral neuromodulation to promote analgesia in pain areas, using two transcutaneous self-adhesive electrodes Axelgaard 5 cm x 5 cm with 2 cm of distance between them. The parameters used were frequency = 100 Hz, pulse width = 50-100 μs, and current intensity according to the patient's sensitivity.

Postural group was treated with biofeedback, manual therapy, and postural exercises, which promoted pelvic mobility and functional training associated with respiratory exercises increasing the diaphragmatic excursion. Postural exercises consisted of 10 repetitions of breathing exercises in the lay-down position, 10 repetitions of hip anteversion and retroversion in the sitting position, and 10 repetitions of hip anteversion, retroversion, and lateral movement in the stand-up position.

The biofeedback and TENS device used was a Myotrac Infiniti T9800 (Thought Technology Ltda., Montreal, Canada, ISO 13485:2016ISO 13485:2016), a 2-channel system of surface electromyography and electrostimulation using the Biograph Infiniti platform. For biofeedback training, we used intravaginal electrodes St-Cloud/Femelex 6.9 cm.

All participants were evaluated post treatment and at 3 months of follow-up using the same procedures of the initial assessment.

All participants were instructed to perform home training daily 3 times/day during treatment and completed an exercise diary to demonstrate adherence to treatment. TENS and Conventional groups were instructed to perform pelvic floor relaxation exercises, and Postural group was instructed to perform pelvic floor relaxation exercises plus postural exercises.

### Sample calculation and statistical analysis

The sample size analysis was calculated in a pilot study based on previous nonpublished data of our participants (n=15). The variables considered in the sample size calculation included “Perineal and Suprapubic Pain VAS Score” and “O’Leary-Sant – Symptoms and Problems Index”. The “O’Leary-Sant – Problems Index” was chosen to estimate the sample size of this study because it reached the largest number of patients. The estimated sample was 27. Considering 20% loss, the calculated sample size was 22 volunteers. The sample sizes calculated for other variables were 12 for perineal pain and 18 for suprapubic pain and urinary symptoms. G Power software, version 3.1, was used with the following parameters: MANOVA Test for repetitive measures to compare the three groups, α=0.005, power (1-β) =0.80 and effect size (f): 0.55

Statistical analysis was performed using the SPSS program (IBM, SPSS Statistics for Windows, Version 21.0. Armonk, NY: IBM Corp.). To assess the normality of continuous variables, the Kolmogorov–Smirnov test was used. The Z score was used to standardize data that did not follow the normality curve.

Descriptive analysis was used (mean ± standard deviations) for the characterization of the groups. For the analysis of continuous variables, the univariate general linear model (GLM) test was used to compare the groups. The analysis of categorical variables was performed using the chi-square test and, when necessary, Fisher's test.

The GLM for repeated measures was performed for the treatment. The significance level was α ≤ 0.05 (24).

## RESULTS

**Figure 1:**
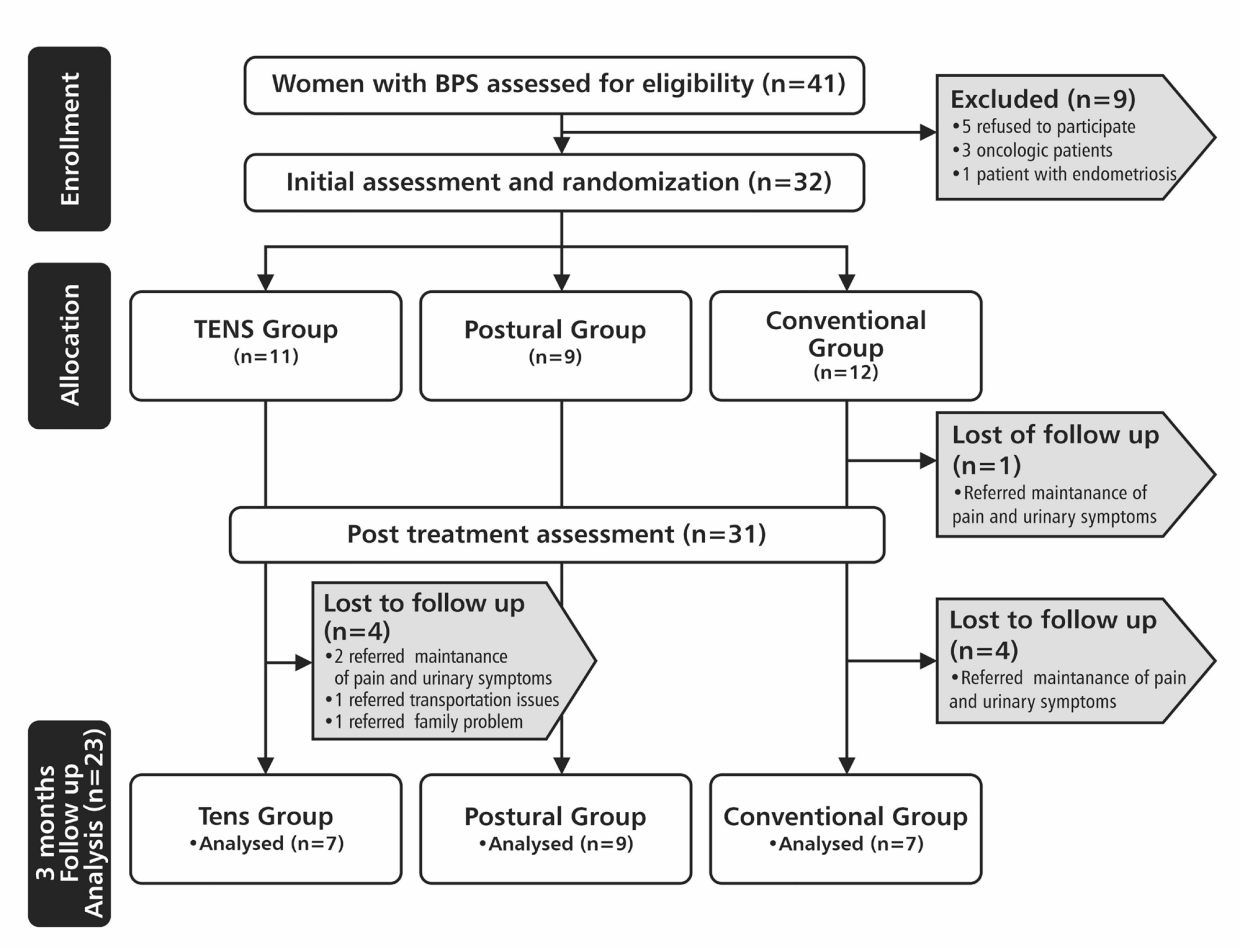
Flowchart of the study.

Of 41 women with BPS initially assessed by eligibility, 9 were excluded. Thus, 32 women were randomized into three groups of treatment: TENS Group, Postural Group and Conventional Group. The study flowchart is shown in Figure-1. The socio-demographic characteristics of 23 women (mean age = 51.95 ± 11.55 and BMI= 26.57 ± 4.23) who completed the 3 months of follow-up are shown in Table-1.

**Table 1:** Sociodemographic data of the sample (n=23).

		TENS (n=7)	Postural (n=9)	Conventional (n=7)	p value
Age (years ± SD)		54±13	48±11	54±9	0.43
BMI (Kg/cm2 ± SD)		24.0 ± 4.0	28.8 ± 3.9	26.3 ± 3.7	0.068
**Education level N (%)**					
	Illiterate	1 (14.3%)	3 (33.3%)	1 (14.3%)	0.452
	Elementary school	1 (14.3%)	1 (11.1%)	2 (28.6%)	
	High school	2 (28.6%)	4 (44.4%)	4 (57.1%)	
	University	1 (14.3%)	1 (11.1%)	0 (0%)	
	Postgraduate	2 (28.6%)	0 (0%)	0 (0%)	
**Marital status N (%)**					
	Single	2 (28.6%)	4 (44.4%)	3 (42.9%)	0.664
	Married	2 (28.6%)	4 (44.4%)	1 (14.3%)	
	Divorced	2 (28.6%)	0 (0%)	2 (28.6%)	
	Widow	1 (14.3%)	1 (11.1%)	1 (14.3%)	
Menopause N (%)		5 (71.4%)	4 (44.4%)	6 (85.7%)	0.209
Time of symptoms (years)		6.60 ±5.20	21.70±15.90	9.40±6.50	**0.028**
Use of painkillers N (%)		6 (85.7%)	9 (100%)	6 (85.7%)	0.531
VAS Perineal pain score (0-10)		7.28±3.86	7.88±1.16	6.14±3.13	0.479
VAS Suprapubic pain score (0-10)		7.57±3.55	6.11±3.58	6.00±2.88	0.621
Urinary Symptoms (ICSI) (0-20)		12.00±6.83	16.66±3.28	15.14±2.73	0.146
Urinary Problems (ICPI) (0-16)		9.43±6.05	13.44±4.47	14.43±2.76	0.121
Sexual function (FSFI) (2-36)		7.48 ± 8.15	12.58 ± 9.54	10.47± 10.55	0.34

**SD** = standard deviation; **BMI** = Body Mass Index; **VAS** = Visual Analogue Scale; **ICSI** = Interstitial Cystitis Symptom Index; **ICPI** = Interstitial Cystitis Problem Index; **FSFI** = Female Sexual Function Index.

### Perineal and Suprapubic Pain

Postural group had a significantly longer time of symptoms than the other two groups (p=0.028) (Table-1).

After treatment, Postural group exhibited significantly improved perineal and suprapubic pain compared to pretreatment assessment (p<0.001 and p=0<0.001, respectively) and the suprapubic improvement persisted during follow-up (p=0.001).

The comparison between groups showed that Postural group exhibited significantly improved perineal and suprapubic pain after treatment (p=0.002 and p=0.026, respectively) and during follow-up compared to Conventional group (p=0.008 and p=0.011, respectively).

### Urinary Symptoms (ICSI) and Urinary Problems (ICPI)

Postural group showed significant improvement in urinary symptoms and problems after treatment compared to pretreatment (p<0.001 and p=0.005, respectively) and a persistence of improvement during follow-up (p<0.001 and p=0.001, respectively).

The comparison between groups showed that TENS group exhibited significantly improved urinary symptoms problems after treatment compared to Conventional group (p=0.043 and p=0.048, respectively). During the follow-up, Postural group showed a significant improvement in urinary symptoms and problems compared to Conventional group (p=0.017 and p=0.025, respectively). Table-2 shows the intragroup and between-group comparisons of perineal and suprapubic pain, urinary symptoms, and problems (ICSI and ICPI, respectively) at pretreatment and posttreatment as well as during follow-up. We observed that conventional treatment alone did not achieve satisfactory results.

**Table 2:** Pain and urinary symptoms' ratings pretreatment, post treatment and during follow-up.

Variable		Pre treatment	Post treatment	Follow-up	p value intra groups			p value inter groups		
					Pre x Post	Post x Follow-up		Pre treatment	Post treatment	Follow-up
**Perineal Pain VAS Score (0-10)**										
	TENS	7.30±3.90	3.30±3.40	3.45±4.40	<0.001 ↑	0.002 ↓	TENS X Postural	0.676	0.179	0.214
	Postural	7.90±1.20	1.35± 2.00	1.44±1.74	<0.001 ↑	<0.001 ↓	TENS X Conventional	0.457	0.057	0.132
	Conventional	6.15±3.15	6.30±3.00	6.00±2.80	0.853	0.898	Postural X Conventional	0.234	0.002	0.008
**Suprapubic Pain VAS Score (0-10)**										
	TENS	7.60±3.55	2.60±3.35	3.30±3.70	<0.001 ↑	0.001 ↓	TENS X Postural	0.402	0.826	0.398
	Postural	6.11±3.60	2.22±2.90	1.90±3.00	0.001 ↑	0.001 ↑	TENS X Conventional	0.395	0.052	0.082
	Conventional	6.00±2.90	6.00±3.10	6.42±2.93	1.00	0.71	Postural X Conventional	0.949	0.026	0.011
**Urinary Symptoms (ICSI) (0-20)**										
	TENS	12.00±6.85	9.14±8.00	10.00±7.80	0.07	0.28	TENS X Postural	0.054	0.734	0.492
	Postural	16.70±3.30	10.10±5.00	8.10±4.80	<0.001 ↑	<0.001 ↑	TENS X Conventional	0.209	0.043	0.088
	Conventional	15.15±2.75	15.60±2.60	15.15±2.05	0.77	1.00	Postural X Conventional	0.512	0.066	0.017
**Urinary Problems (ICPI) (0-16)**										
	TENS	9.50±6.05	8.00±7.10	9.00±7.45	0.286	0.805	TENS X Postural	0.100	0.506	0.524
	Postural	13.45±4.50	9.80±5.20	7.20±5.60	0.005 ↑	0.001 ↑	TENS X Conventional	0.056	0.048	0.111
	Conventional	14.45±2.80	13.85±2.00	13.85±1.35	0.666	0.742	Postural X Conventional	0.677	0.136	0.025

**VAS** = Visual Analogue Scale; **ICSI** = Interstitial Cystitis Symptom Index; **ICPI** = Interstitial Cystitis Problem Index;

↑ = improved;

↓ = worsened

Related to the presence of perineal and suprapubic trigger points, we observed that Postural group showed a significant decrease after treatment and during follow-up (Table-3).

**Table 3:** Comparison between the presence of perineal and suprapubic trigger points between groups at pretreatment, post treatment and during follow-up.

Trigger points	Groups	Pretreatment n (%)	Post treatment n (%)	Follow-up n (%)	p value
Perineal area	TENS (n=7)	6 (85.7%)	4 (57.1%)	3 (42.8%)	0.087
	Postural (n=9)	9 (100%)	4 (44.4%)	4 (44.4%)	**0.009** *
	Conventional (n=7)	6 (85.7%)	6 (85.7%)	6 (85.7%)	1.0
Suprapubic area	TENS (n=7)	6 (85.7%)	3 (42.8%)	4 (57.1%)	0.088
	Postural (n=9)	7 (77.8%)	5 (55.5%)	3 (33.3%)	**0.039** *
	Conventional (n=7)	6 (85.7%)	6 (85.7%)	6 (85.7%)	1.0

* p value was significant in comparison between pretreatment versus post treatment assessment.

Related to medications, we did not observe a decrease in anticholinergic, antidepressant, or anticonvulsant intake after treatment. However, we observed a significant decrease in painkiller intake in Postural Group after treatment. Regarding sexual function, none of the groups presented any improvement after treatment (Table-4).

**Table 4:** Comparison of painkiller's intake and sexual function score (FSFI) between groups at pretreatment and post treatment.

Painkiller's intake	Pretreatment	Post treatment	P value
TENS n (%)	6 (85.7%)	6(85.7%)	1.0
Postural n (%)	9 (100%)	5 (55.5%)	0.035
Conventional n (%)	6(85.7%)	6(85.7%)	1.0
**FSFI**			
TENS (mean ± SD)	7.48 ± 8.15	7.60 ± 7.85	0.85
Postural (mean ± SD)	12.58 ± 9.54	14.74 ± 13.82	0.26
Conventional (mean ± SD)	10.47± 10.55	13.57 ± 14.92	0.87

FSFI = Female Sexual Function Index (2-36); SD = standard deviation.

In each session, the physiotherapists asked the participants about possible side effects caused by the treatment, such as burning sensation, pain, and discomfort. We noticed that no women from the whole sample reported any complaints during or after sessions during the entire treatment period.

## DISCUSSION

Biofeedback and manual therapy are recommended in the main urology guidelines as conventional treatment for patients with BPS; however, a common protocol is not available. We considered these techniques as conventional treatment for research purposes.

In this randomized controlled trial, we compared conventional treatment alone with the combination of TENS or postural exercises to verify whether the combination would be beneficial.

Our study showed significant improvement of perineal and suprapubic pain by the association of postural exercises with conventional treatment. In a randomized control trial with 81 women with BPS, Fitzgerald et al. (11) showed that 59% of women treated with manual therapy reported a subjective improvement of pain and pelvic floor tension. However, in our study, we did not observe that this conventional treatment improved perineal and suprapubic pain in women with BPS.

In this study, Postural group showed a significant improvement in urinary symptoms and problems after treatment and during follow-up. Haugstad et al. (19) evaluated 60 women with chronic pelvic pain to study the clinical characteristics of posture, movement, gait, sitting posture, and respiration and demonstrated a nonfunctional motor pattern and a lack of coordination and irregular high costal respiration. The association of postural exercises, which included pelvic mobility, functional training and diaphragmatic exercises, was applied in participants in the postural group to improve global movements, coordination, and balance due to a protective blockage of movement to avoid pain. This blockage decreases diaphragm excursion and pelvic mobility and leads to an increase in pelvic floor and lumbopelvic tension, stiffening of the core and consequently increased pain intensity. Nickel et al. (25) observed in 93 women with BPS that approximately 50% of patients experienced clinically significant improvement using an individualized phenotype-directed therapeutic approach. Our approach focusing on postural exercises helped us confirm that technique as a valuable tool for BPS patients with urinary symptoms.

Our study did not show a significant improvement in sexual function. In total, 69.6% of the participants reported menopause symptoms and dysfunctional or absence of sexual activity (65.2%); of these, 52.2% reported dyspareunia and history of sexual abuse (21.7%). In a retrospective study, Seth et al. (26) observed that women with BPS with sexual abuse presented more pain and pelvic tension and fewer urinary symptoms. In our study, we did not observe a significant difference in pain, pelvic tension, or urinary symptoms in women with and without sexual abuse.

The strengths of this study were that it was a randomized prospective controlled trial that used conventional treatment associated with postural exercises with a significant improvement in perineal and suprapubic pain and urinary symptoms. The treatment consisted of 10 sessions once weekly with no side effects reported by the participants. Postural group showed increased adherence during the treatment and during follow-up (100%) compared to TENS and Conventional groups (63.6% each group). We concluded that postural exercises were easily understood by the participants, promoting their adherence.

This study had a few limitations. In total, 32 women were randomized and initiated the treatment, and 31 finished it. During follow-up, 23 (74.19%) remained in the study. We calculated a loss of approximately 20% out of 23 and not out of 32. If so, our study had an approximately 30% attrition rate. Although the sample size was small for a randomized controlled trial, the results showed a positive and impactful improvement.

## CONCLUSIONS

Biofeedback and manual therapy associated with postural exercises showed a significant improvement in perineal and suprapubic pain and urinary symptoms after treatment and during follow-up. Both results suggest a possible role for the use of this physiotherapy technique to treat BPS patients. Longer follow-up and a larger number of patients are necessary to confirm these conclusions.
